# Temporomandibular Disorders and Related Factors in a Group of Iranian Adolescents: A Cross-sectional Survey

**DOI:** 10.5681/joddd.2011.028

**Published:** 2011-12-19

**Authors:** Masoumeh Ebrahimi, Hossein Dashti, Maryam Mehrabkhani, Mohammad Arghavani, Avideh Daneshvar-Mozafari

**Affiliations:** ^1^Dental Research Center, Mashhad University of Medical Sciences, Mashhad, Iran; ^2^Assistant Professor, Department of Pediatric Dentistry, Dental School, Mashhad University of Medical Sciences, Mashhad, Iran; ^3^Assistant Professor, Department of Prosthodontics, Dental School, Mashhad University of Medical Sciences, Mashhad, Iran; ^4^Dentist, Private Practice, Mashhad, Iran

**Keywords:** Adolescent, epidemiology, temporomandibular joint disorders

## Abstract

**Background and aims:**

Temporomandibular disorders (TMDs) are the most common condition affecting the tem-poromandibular joint and associated structures. The aim of this study was the epidemiologic evaluation of TMDs and re-lated factors in a group of Iranian adolescents.

**Materials and methods:**

This descriptive cross-sectional survey included a sample of800 high school students (400 girls and 400 boys) aged 14 to18 years, in Mashhad, Iran, selected using cluster sampling. Examiners completed question-naires and performed the clinical examinations. Data were analyzed with the Chi-square and Fisher exact tests.

**Results:**

The prevalence of TMDs in the studied sample was 34.7%. The most common signs of TMDs were clicking, muscle tenderness and TMJ tenderness. The most prevalent predisposing factors of TMDs were clenching, premature con-tact in protrusive movement and bruxism. A clear predominance was seen in girls (40.5%) compared with boys (29%) (P = 0.001).

**Conclusion:**

Signs and symptoms of TMDs were prevalent in Iranian adolescents with a clear female predominance.

## Introduction

Temporomandibular disorders (TMDs) are defined as signs and symptoms in the temporomandibular joint (TMJ), muscles of mastication and related structures.^1^ A multifactorial disease, TMD has a controversial etiology.^2^ It results from psychological factors,^3 , 4^malocclusion,^5 , 6^ oral parafunction,^5 , 7^oral habits,^5 , 8^trauma,^5 , 9^ occlusion,^5 , 10^ and stress.^5 , 9^ A variety of signs and symptoms, such as pain—particularly in the muscles of mastication, muscular dysfunction, TMJ sounds (clicking and crepitus), headaches, earaches and occlusal dysfunction have been attributed to TMDs.^5^ The prevalence of TMDs has been reported to be from 6% to 68% in various studies,^11^mostly because of differences in diagnostic methods and the type of the study. Aging and different clinical situations also play important roles in the severity of its signs and symptoms.^3^In the study of Casanova-Rosado et al,^12^ gender, bruxism, stress, unilateral chewing and tooth loss were the most important factors associated with TMDs in adults.^12^ It has been shown that gender and low self-confidence, compared with dental factors, have a significant effect on TMDs in adolescents.^13^ In addition, unilateral chewing and clenching can increase the risk of pain, TMJ sounds and limitation of jaw movement.^14^

As late diagnosis of TMDs may result in irreversible and destructive effects on the TMJ, its early evaluation plays a crucial role in the treatment process.^15 , 16^ In addition, increasing public need for comprehensive oral health care has resulted in a higher demand for TMDs treatment; therefore, epidemiological information including rate, distribution and etiology of TMDs in the population is valuable. In Iran, however, there are spare data on the epidemiology of TMDs. Basafa and Shahabee^17^ have reported a prevalence of 22.1% for TMDs among Iranian medical and dental students. Therefore, we carried out the present study to epidemiologically evaluate the signs, symptoms and related factors of TMDs in a group of Iranian adolescents.

## Materials and Methods

This descriptive cross-sectional study was done on a sample of high school students (400 girls and 400 boys) selected from seven districts of Mashhad, Iran, using the cluster sampling method. This study was approved by the Research Ethics Committee of Mashhad University of Medical Sciences (No. 86743). Informed consent was given by the parents before the inclusion in the study.

The girls were examined by a female dentist, and the boys were examined by a senior dental student. Before the study, the examiners took part in a clinical calibration exercise with the principal investigators. After 20 adolescents were examined twice for reliability, agreement between the first and second examinations was found to be good. The examiners completed questionnaires and performed the clinical examination. The questionnaires were then evaluated by two pediatric dentists and a prosthodontist and found to be valid. The questionnaire included items of patient history: ear, nose and throat (ENT) infections; oral trauma; orthodontic treatment; and headache. The muscles of mastication were examined clinically, and the TMJ (including joint sounds and pain) and related etiological factors of TMDs were evaluated: malocclusion, premature contacts and parafunctional habits.

The data were analyzed with the Chi-square and Fisher exact tests. Multivariate analysis was performed using stepwise logistic regression. Statistical significance was based on probability values of <0.05.

## Results

The prevalence of TMDs was 34.7% in the studied group of adolescents. Data revealed a clear predominance in girls (40.5%) compared with boys (29%) (P = 0.001). The frequency of clicking, myofascial pain and TMJ pain in subjects with TMDs was 74.1%, 67.6% and 35.2%, respectively. Table 1 shows the frequency of ENT infections, oral trauma, orthodontic treatment and headache history. There was a significant difference between subjects having a history of ENT infections with and without TMDs (P = 0.001). However, there was no significant difference between subjects with or without TMDs having oral trauma, orthodontic treatment and headache histories.

**Table 1:** Frequency distribution of ear, nose and throat (ENT) infection, trauma, orthodontic treatment, and headache in the studied population (n = 800)

History	TMD+	TMD-	Total	P-value
ENT infection	34.3%	26%	28.9%	P=0.001*
Trauma	20.4%	11.1%	14.3%	P>0.05
Orthodontic treatment	5.5%	4.1%	4.6%	P>0.05
Headache	14.1%	12.1%	12.8%	P>0.05

TMD+, with temporomandibular disorders; TMD−, without temporomandibular disorders; ENT infection, ear, nose, and throat infection. * Statistically significant.

### Malocclusion

**Figure1:**
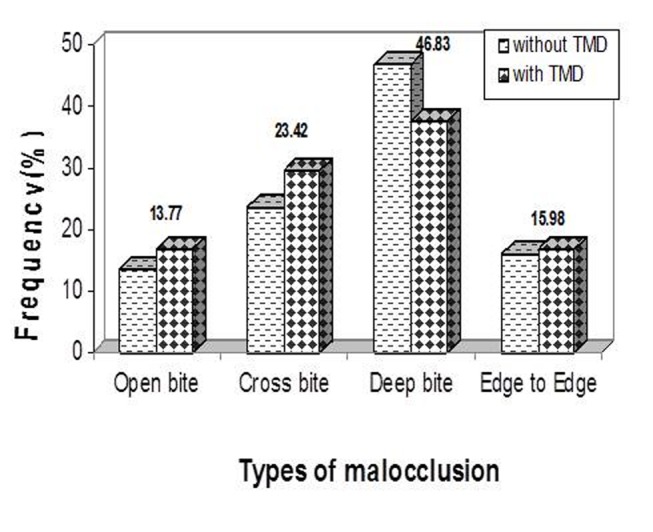
Types of malocclusion in the studied population.

Malocclusion was found in 65.1% of the subjects. According to the data, the role of malocclusion (66.2% in subjects with and 64.4% in those without TMDs) was not significant. Deep bite malocclusion was the most frequently associated condition (Figure 1).

### Premature contacts

**Figure 2:**
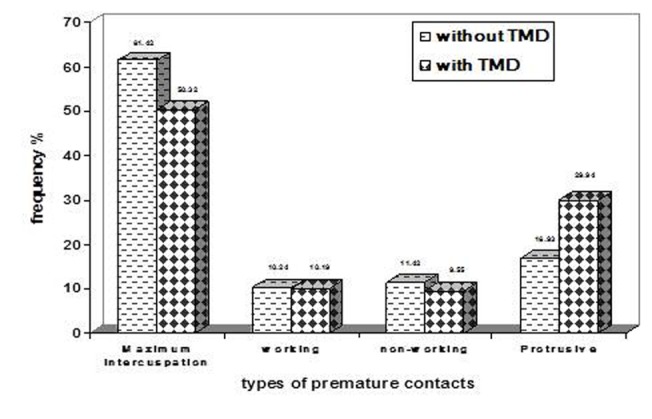
Types of premature contacts in the studied population.

Premature contacts were seen in 35.7% of the study group (39.9% in subjects with TMDs and 33.6% in those without). The occlusion was evaluated in maximum intercuspation and in working, nonworking and protrusive movements. Those who had TMDs showed significant premature contacts in protrusive movement and maximum intercuspation (P = 0.000). The highest frequency of premature contacts was seen in maximum intercuspation in both subgroups (Figure 2).

### Parafunctional habits

**Figure 3:**
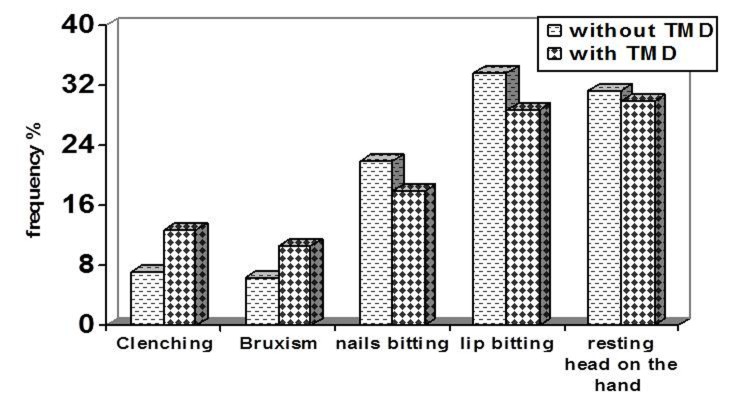
Types of parafunctional habits in the studied population.

Of all studied subjects, 68.1% had parafunctional habits (70.5% of subjects with TMDs and 66.8% of those without), and there was no significant difference between those subgroups (P = 0.301). Excepting nail and lip biting and resting head on the hand as parafunctional habits, clenching and bruxism had a significant relation to TMDs (P = 0.001). Lip biting and resting head on the hand were the most frequent habit in both subgroups (Figure 3).

The results of logistic regression showed that clenching was the most important predisposing factor in TMDs. Statistically, the possibility of TMDs in people with clenching was 2.6 times that of the population as a whole. Premature contacts in protrusive movement and bruxism followed in frequency (Table 2).

**Table 2:** Stepwise logistic regression analysis of parafunctional habits among subjects with temporomandibular disorders in the studied population

Variables	Regression coefficient	Odds ratio	P-value	Confidence interval
Clenching	0.981	2.667	<0.05*	1.538–4.624
Premature contacts	0.879	2.41	<0.05*	1.533–3.788
Bruxism	0.607	1.834	0.033	1.049–3.208
Gender	-0.402	0.669	0.012	0.489–996

Premature contacts during protrusive movements were considered. * Statistically significant.

## Discussion

This study evaluated the signs, symptoms and related etiologic factors of TMDs in a group of high school students in Iran. The prevalence of TMDs was found to be 34.7%, which is lower than those found in similar studies in an Italian population (54.3%),^18^ Mexican adolescents (46.1%),^12^Brazilian adolescents,^1^ or the American population,^19^ and higher than those found in Japanese^20^or Brazilian adolescents.^21^ Joint pain was seen in 35.2% of the studied group, which is more than that in Japanese adolescents (16%).^14^ Also in the present survey, 67.6% of subjects with TMDs suffered from pain in the muscles of mastication, which McDonald defined as a TMDs symptom.^22^

Of all subjects in our study, 74.1% showed clicking, which is more than that of Japanese adolescents,^14^Brazilian adolescents^23^or of Iranian dental students in another study.^17^ It has been reported that joint sounds are diagnosed as one of the most prevalent symptoms in children.^13 , 15^ Our results also showed that the proportion of females with TMDs (40.5%) was significantly higher than that of the male subgroup (29%), in agreement with several other studies. ^1,18,21,24 - 27^ Some further studies have, however, shown no differences between genders,^23 , 28^ and Motegi et al^20^ concluded that the gender factor does not have any significant effect on TMDs. In other words, if there is any difference between sexes, it is because women tend to have check-ups more frequently than do men.^20^ In this regard, hormonal differences of genders have also been suggested as an important factor in TMDs. ^20^

Our findings showed that deep bite, open bite, crossbite and edge-to-edge malocclusions did not have any significant associations with TMDs; this was also concluded in several other studies.^16 , 17 , 21^ Despite these results, there are studies that indicate different types of malocclusions have a significant effect on TMDs.^16 , 20^ Except lip and nail biting and resting the head on the hand as parafunctional habits, clenching, premature contacts during protrusive movements, and bruxism had a significant association with TMDs. The relationship between parafunctional habits and TMDs was also demonstrated previously,^19^ although several studies found no such relationship. ^16, 21, 24, 29, 30^

According to the results of this survey, TMDs are prevalent among Iranian adolescents, with a clear female predominance. TMDs decrease the quality of life^31^and pose treatment challenges for the dentists. Therefore, every effort must be made for prevention and early diagnosis of TMDs. Raising public awareness of the issue especially in schools among the adolescents and their parents by means of pamphlets and other media should be considered.

## References

[1] de oliveira as, dias em, contato rg, berzin f (2006). prevalence study of signs and symptoms of temporomandibular disorder in brazilian college students. braz oral res.

[2] dimitroulis g (1998). temporomandibular disorders: a clinical update. bmj.

[3] otuyemi od, owotade fj, ugboko vi, ndukwe kc, olusile oa (2000). prevalence of signs and symptoms of temporomandibular disorders in young nigerian adults. j orthod.

[4] rollman gb, gillespie jm (2000). the role of psychosocial factors in temporomandibular disorders. curr rev pain.

[5] okeson jp (2008). management of temporomandibular disorders and occlusion.

[6] hagag g, yoshida k, miura h (2000). occlusion, prosthodontic treatment, and temporomandibular disorders: a review. j med dent sci.

[7] feteih rm (2006). signs and symptoms of temporomandibular disorders and oral parafunctions in urban saudi arabian adolescents: a research report. head face med.

[8] castelo pm, gaviao mb, pereira lj, bonjardim lr (2005). relationship between oral parafunctional/nutritive sucking habits and temporomandibular joint dysfunction in primary dentition. int j paediatr dent.

[9] greenberg m, glick m, ship j (2008). burket’s oral medicine.

[10] de boever ja, carlsson ge, klineberg ij (2000). need for occlusal therapy and prosthodontic treatment in the management of temporomandibular disorders part i occlusal interferences and occlusal adjustment. j oral rehabil.

[11] poveda roda r, bagan jv, diaz fernandez jm, hernandez bazan s, jimenez soriano y (2007). review of temporomandibular joint pathology part i: classification, epidemiology and risk factors. med oral patol oral cir bucal.

[12] casanova-rosado jf, medina-solis ce, vallejos-sanchez aa, casanova-rosado aj, hernandez-prado b, avila-burgos l (2006). prevalence and associated factors for temporomandibular disorders in a group of mexican adolescents and youth adults. clin oral investig.

[13] godoy f, rosenblatt a, godoy-bezerra j (2007). temporomandibular disorders and associated factors in brazilian teenagers: a cross-sectional study. int j prosthodont.

[14] miyake r, ohkubo r, takehara j, morita m (2004). oral parafunctions and association with symptoms of temporomandibular disorders in japanese university students. j oral rehabil.

[15] pinkham jr (2005). pediatric dentistry: infancy through adolescence.

[16] barone a, sbordone l, ramaglia l (1997). craniomandibular disorders and orthodontic treatment need in children. j oral rehabil.

[17] basafa m, shahabee m (2006). prevalence of tmj disorders among students and its relation to malocclusion. the iranian journal of otorhinolaryngology.

[18] ciancaglini r, radaelli g (2001). the relationship between headache and symptoms of temporomandibular disorder in the general population. j dent.

[19] nassif nj, al-salleeh f, al-admawi m (2003). the prevalence and treatment needs of symptoms and signs of temporomandibular disorders among young adult males. j oral rehabil.

[20] motegi e, miyazaki h, ogura i, konishi h, sebata m (1992). an orthodontic study of temporomandibular joint disorders part 1: epidemiological research in japanese 6-18 year olds. angle orthod.

[21] pereira lj, pereira-cenci t, del bel cury, pereira sm, pereira ac, ambosano gm, et al (2010). risk indicators of temporomandibular disorder incidences in early adolescence. pediatr dent.

[22] mcdonald re, avery dr (2011). dentistry for the child and adolescent.

[23] bonjardim lr, gaviao mb, pereira lj, castelo pm, garcia rc (2005). signs and symptoms of temporomandibular disorders in adolescents. braz oral res.

[24] barbosa tde s, miyakoda ls, pocztaruk rde l, rocha cp, gaviao mb (2008). temporomandibular disorders and bruxism in childhood and adolescence: review of the literature. int j pediatr otorhinolaryngol.

[25] hobson ka, huang gj, covell da,  jr  jr (2008). patterns of dental care utilization among patients with temporomandibular disorders. j orofac pain.

[26] nilsson im, list t, drangsholt m (2007). incidence and temporal patterns of temporomandibular disorder pain among swedish adolescents. j orofac pain.

[27] liljestrom mr, le bell y, laimi k, anttila p, aromaa m, jamsa t, et al (2008). are signs of temporomandibular disorders stable and predictable in adolescents with headache. cephalalgia.

[28] pow eh, leung kc, mcmillan as (2001). prevalence of symptoms associated with temporomandibular disorders in hong kong chinese. j orofac pain.

[29] gavish a, halachmi m, winocur e, gazit e (2000). oral habits and their association with signs and symptoms of temporomandibular disorders in adolescent girls. j oral rehabil.

[30] seraj b, ahmadi r, mirkarimi m, ghadimi s, beheshti m (2009). temporomandibular disorders and parafunctional habits in children and adolescents: a review. journal of dentistry, tehran university of medical sciences.

[31] naito m, yuasa h, nomura y, nakayama t, hamajima n, hanada n (2006). oral health status and health-related quality of life: a systematic review. j oral sci.

